# Evaluation of genetic diversity within different rabbit (*Oryctolagus cuniculus*) genotypes utilizing start codon targeted (SCoT) and inter-simple sequence repeat (ISSR) molecular markers

**DOI:** 10.5194/aab-67-285-2024

**Published:** 2024-07-02

**Authors:** Nouran Elsayed, Abd Elrahman E. Mandour, Mamdouh K. A. Amin, Fayiz M. Reda, Heba S. A. Taha, Alessandro Di Cerbo, Mahmoud M. Azzam, Mahmoud Alagawany

**Affiliations:** 1 Genetics Department, Faculty of Agriculture, Zagazig University, Zagazig, Egypt; 2 Poultry Department, Faculty of Agriculture, Zagazig University, Zagazig, Egypt; 3 School of Biosciences and Veterinary Medicine, University of Camerino, Matelica, Italy; 4 Department of Animal Production, College of Food & Agriculture Sciences, King Saud University, Riyadh 11451, Saudi Arabia

## Abstract

This work aimed at studying the genetic diversity among different rabbit genotypes reared in Egypt by two different molecular markers (start codon targeted, SCoT, and inter-simple sequence repeat, ISSR) to improve breeding strategies. Five different groups of rabbits were used Gabali (Gab), New Zealand white (NZW), Californian (Cal), Rex, and Papillon (Pap). DNA was extracted and analyzed using SCoT and ISSR-PCR, and the obtained fragments were analyzed. Six SCoT primers scored 60 bands with 78.33 % polymorphism; primer SCoT 6 was the most polymorphic marker with 92.31 % polymorphism, while SCoT 5 was the lowest with 60 %. A dendrogram based on SCoT-PCR revealed that the Rex breed was the most genetically different. Seven ISSR primers gained 56 bands in total with 49.762 % polymorphism. ISSR 4 was the most polymorphic primer that detected 75 % of polymorphism, while ISSR 6 was not able to detect any polymorphism. It was suggested that the SCoT markers may be more effective than ISSR for differentiating and identifying the genetic variations within investigated breeds. Also, the usage of molecular markers of SCoT and ISSR may be more proper for calculating genetic diversity and common ancestry among tested rabbit breeds. Furthermore, evaluating genetic variability is important for enhancing existing breeds' adaptation to ecological alterations and crucial for preservation or breeding purposes.

## 1 Introduction

Rabbits (*Oryctolagus cuniculus*), live in various regions across the globe and possess a distinctive set of characteristics, including, but not limited to, exceptional productivity and advantageous attributes. Rabbits provide excellent meat for human consumption and may play a significant role in solving a part of the meat shortage (Elwan et al., 2019). From the 6th century, people have tamed rabbits to benefit from their meat and fur. These docile and adaptable creatures possess a temperament that lends itself well to human interaction and scientific study, and their overall health and wellbeing surpasses that of comparable animals, such as rats and mice (Houdebine and Fan, 2009; El-Sabrout and Aggag, 2017). Rabbits possess unique phenotypic variations and significant commercial and societal importance as production animals (Carneiro et al., 2014). Several rabbit breeds in Egypt are at risk of extinction because of decreased population numbers, with some being already extinct (Khalil and Baselga, 2002).

Studying animal genetic diversity using molecular markers is important for several reasons. Molecular markers allow for the identification of genetic variation between individuals and can be used to detect the genetic potential of an animal before its expression as a phenotype (Nam et al., 2020). They provide important information about selection programs and genetic improvement and can be utilized for MAS selection in breeding strategies (Perera et al., 2020). Molecular markers, such as single nucleotide polymorphisms (SNPs), are abundant in the genome and can be used for genetic analysis, paternity tests, and association with phenotypes (da Silva Reis et al., 2018). They also allow for the mapping of genes and quantitative trait loci (QTL) that influence production characteristics (McLennan et al., 2019). Additionally, molecular markers can be used to evaluate population-level genetic diversity and inform conservation decision-making (Perera and Manimekalai, 2021). Overall, molecular markers could be used as important methods for understanding and managing animal genetic diversity.

SCoT markers were used in several studies to measure genetic diversity in different animal species, such as fish species of the family Moronidae (Abu Almaaty et al., 2020), *Erodium* species (Shahram et al., 2021), *Prunus sibirica* populations in Inner Mongolia (Buer et al., 2022), and *Elymus sibiricus* (Zhang et al., 2015). These studies demonstrate the utility of SCoT markers in assessing genetic diversity and relationships in animals.

Genetic variation is considered an essential role in enhancing the stocks of pastoralists and farmers by enabling them to adjust their livestock populations in response to environmental circumstances and evolving needs (Sastry, 2023). Breeders require genetic variability to enhance existing breeds' ability to adapt to ecological alterations and spread epidemics (Galal et al., 2013). The evaluation of genetic differences in a species is crucial for the preservation or breeding purposes (Raheimi et al., 2005).

The initial step in safeguarding genetic resources and preventing animal germplasm erosion is genetic characterization. Evaluating genetic variation within breeds is a vital step in managing animal biodiversity, especially in detecting genetically distinct structures (Eltanany et al., 2015). Kettenring et al. (2014) proposed that the adaptability of thousands of domesticated species to various environmental factors such as climate, epidemic, features of the soil, nutrition provenance, and geographical conditions has been facilitated by their genetic diversity. Genetic diversity among populations is crucial for maintaining ecological functioning and providing vital benefits for humans (Hollingsworth et al., 2020).

The utilization of genetic data facilitates the preservation of endangered species and their management(Allendorf and Luikart, 2007). Ben Larbi et al. (2012) showed that genetic data can help in creating appropriate preservation and breeding strategies, as it informs domestication and evolution history. It also facilitates studies linking phenotypes and genotypes, and these variations indicate genetic diversity in their gene pool (M. A. Badr et al., 2016; O. A. Badr et al., 2016; Hoban et al., 2022). Great improvement has occurred in animal genetics in relation to DNA-based genetic markers. Theoretically, DNA markers can be utilized to detect genetic diversity in the whole genome, as mentioned by Liu and Cordes (2004). Different kinds of molecular techniques were extensively utilized in rabbit diversity studies as RAPD-PCR, PCR-RFLP, ISSR, and microsatellites (Mohamed and Abdelfattah, 2018a; Adeolu et al., 2021).

Collard and Mackill (2009) established a useful marker method, which is the start codon targeted (SCoT) marker. It is a molecular marker of DNA, which targets the species surrounding the start codon (ATG) of genes and their encompassing consensus sequences in a gene family. The SCoT technique employs a singular primer, as noted by Bhattacharyya et al. (2013), and boasts several advantages including, but not limited to, simple primer design, ease of operation, vastly detected polymorphism, cheap cost, excellent reproducibility, and significant association with phenotypic data (Chen et al., 2009). These markers have proven useful in DNA fingerprinting, population construction analysis, cultivar identification, marker-assisted selection, and SCAR marker development, as well as the study of quantitative trait loci and phylogenetic relationships within diverse plant species (Gorji et al., 2012; Yang et al., 2019; Cabo et al., 2014; Zhang et al., 2015; Jalilian et al., 2018).

The inter-simple sequence repeat (ISSR) marker has been extensively utilized in various applications, such as those related to the identification of genetic enhancement and the development of species-specific markers. This particular approach facilitates researchers to distinguish between various species even in the absence of comprehensive DNA sequence information (Ng and Tan, 2015; Holliday et al., 2018; Karsli et al., 2020; Xia et al., 2021). There is limited information available regarding the genetic diversity between different rabbit genotypes reared in Egypt by two different molecular markers (SCoT and ISSR). Thus, the objective of the current study was to study the genetic diversity and ancestral relations of five rabbit breeds in Egypt at the molecular level through the dual dependence of molecular markers, ISSR, and SCOT to cover different sections of the genome and to improve breeding strategies.

## 2 Materials and methods

### 2.1 Animals, design, and management

This study was performed at the Biochemical Genetics and Molecular Genetics Laboratory of the Genetics Department and the Rabbit Research Farm, Faculty of Agriculture, Zagazig University, Egypt. Examined rabbits were obtained from the farm of the Department of Animal Wealth Development at the Faculty of Veterinary Medicine and the Rabbit Research Farm of the Poultry Department, Faculty of Agriculture, Zagazig University, Zagazig, Egypt.

The experimental materials involved in this study were five different genetic groups of rabbits, including the New Zealand white (NZW) breed, which reared in the United States and is widespread all over the world; the Californian (Cal) breed, developed by crossing Himalayan breeds in southern California; and the Gabali (Gab) breed, a productive local variety in Egypt that has been historically overlooked, without any selection. However, to meet consumer demand, genetic amelioration is necessary to increase meat production (Afifi, 2002; Iraqi et al., 2007). The Rex breed is a domestic breed characterized by its soft, dense fur, which was developed in France, and the Papillon (Pap) rabbit breed was developed in England in the 19th century through selective breeding.

All animals were normal, healthy, and 8 weeks old. Animals were divided into five identical clusters at random (10 rabbits per group). The room where the animals were raised had a wire floor, natural ventilation, and artificial lighting. Animals were brought up in separate enclosures (Galvanized wire; 35cm×50cm×45 cm) that included automated drinkers and feeders. Every animal was raised in the same hygienic and managed conditions for the duration of the experiment. The floor was sanitized every morning to eliminate feces and urine. Water and food were supplied, and fresh water was replenished every day at 09:00 and 15:00 LT. The rabbits were given pelleted food daily at 09:00 LT without fail.

### 2.2 Blood samples collection

A blood sample of approximately 2 mL was obtained from the ear's central arterial vein using suction in centrifuge tubes containing EDTA as an anticoagulant from each animal of the analyzed breeds.

**Table 1:** Sequences, molecular weights, and GC percentage of six start codon targeted primers (SCoT).

Primer	Sequence	MW	GC %
SCoT 2	5 ${}^{\prime }$ CAA CAA TGG CTA CCA CCC-3 ${}^{\prime }$	5397.6	55.56
SCoT 19	5 ${}^{\prime }$ ACC ATG GCT ACC ACC GGC-3 ${}^{\prime }$	5429.6	66.67
SCoT 6	5 ${}^{\prime }$ CAA CAA TGG CTA CCA CGC-3 ${}^{\prime }$	5437.6	55.56
SCoT 3	5 ${}^{\prime }$ CAA CAA TGG CTA CCA CCG-3 ${}^{\prime }$	5437.6	55.56
SCoT 14	5 ${}^{\prime }$ ACG ACA TGG CGA CCA CGC-3 ${}^{\prime }$	5478.6	66.67
SCoT 33	5 ${}^{\prime }$ CCA TGG CTA CCA CCG CAG-3 ${}^{\prime }$	5429.6	66.67

MW: Molecular weight, GC: base content percentage.

### 2.3 Molecular analysis

#### 2.3.1 DNA extraction

A blood sample was used for the extraction of DNA by the non-enzymatic magnetic salting-out method (Suguna et al., 2014).

*SCoT–ISSR PCR analysis.* Six SCoT (Sc) primers (Macrogen Inc., Seoul, Republic of Korea) that were able to amplify DNA fragments were used in this study (Sc 2, Sc 19, Sc 6, Sc 3, Sc 14, and Sc 33). The corresponding sequences of the six primers are provided in Table 1. In order to produce ISSR profiles from rabbit DNA, seven various ISSR markers from Biosearch Technologies (USA) were utilized in the current research (Table 2).

**Table 2:** Seven ISSR primers and their sequences.

Primer	Code	Sequence from 5' to 3'
ISSR 1	ISSR825	5 ${}^{\prime }$ ACACACACACACACT-3 ${}^{\prime }$
ISSR 2	UBC811	5 ${}^{\prime }$ GAGAGAGAGAGAGAGC-3 ${}^{\prime }$
ISSR 3	UBC808	5 ${}^{\prime }$ AGAGAGAGAGAGAGAGC-3 ${}^{\prime }$
ISSR 4	UBC901	5 ${}^{\prime }$ CACACACACACACACARY-3 ${}^{\prime }$
ISSR 5	UBC814	5 ${}^{\prime }$ CTCTCTCTCTCTCTCAT-3 ${}^{\prime }$
ISSR 6	UBC826	5 ${}^{\prime }$ ACACACACACACACACC-3 ${}^{\prime }$
ISSR 7	UBC827	5 ${}^{\prime }$ ACACACACACACACACG-3 ${}^{\prime }$

PCR for each primer was conducted in a 20 µ L final volume divided as follows: 1 µ L of master mix, 1 µ L of primer, and 1 µ L of DNA template, which was then filled up to 20 µ L with distilled water.

*Conditions for the amplification for both SCoT and ISSR-PCR analyses.* The first denaturation process at 94 °C lasted 5 min and was followed by 40 cycles comprising 45 s of denaturation at 94 °C, 1 min at 50–60 °C based on the suitability of the primer, and 3 min of elongation at 72 °C and the final elongation process at 72 °C for 5 min. 1.5 % agarose gel (Sigma Aldrich, Hamburg, Germany; A9414) marked by ethidium bromide was utilized to isolate amplification products and envisaged under UV light in a gel documentation system (bio rad). The acquired bands of these DNA fragments were examined by the GelAnalyzer4software program (Ahmed, 2021) and assembled for each primer in Table 3.

**Table 3:** Results obtained from five different rabbit breeds using six different SCoT primers showing total, monomorphic, polymorphic, and unique bands.

Primer	Total no bands	Monomorphic bands	Polymorphic bands		Polymorphism
			Without unique	Unique	
SCoT 2	10	2	4	4	80 %
SCoT 19	16	3	5	8	81.25 %
SCoT 6	10	3	2	5	70 %
SCoT 3	6	2	2	2	66.67 %
SCoT 14	5	2	1	2	60 %
SCoT 33	13	1	7	5	92.31 %
Total	60	13	21	26	78.33 %

*Statistical analysis.* As variables, PCR patterns were counted across the lanes. The existence of a band of DNA is evaluated as 1, while absence is 0. The collected data were utilized to compute the similarity index and dendrogram using the SPSS 14.0 evaluation version.

**Figure 1:**
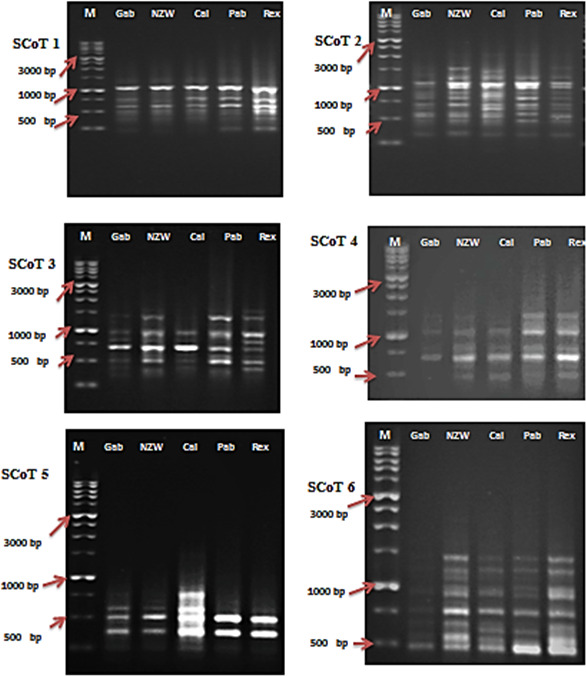
SCoT profiles of the five rabbit breeds as obtained by six SCoT primers lane. SCoT 1: Gabali breeds (Gab), SCoT 2: New Zealand white (NZW), SCoT 3: Californian (Cal), SCoT 4: Papillon (Pap), SCoT 5: Rex (Rex). M =1 Kb marker.

**Figure 2:**
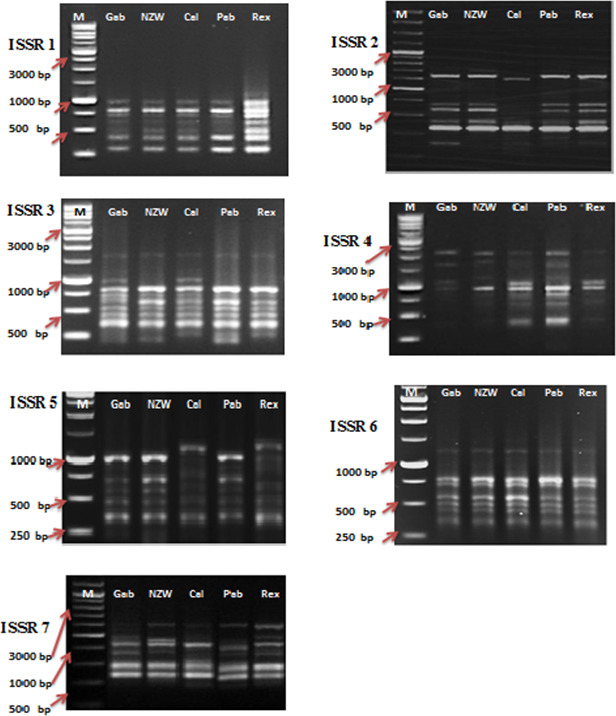
ISSR profiles of the five rabbit breeds as obtained by seven ISSR primers lane. ISSR 1: Gabali breeds (Gab), ISSR 2: New Zealand white (NZW), ISSR 3: Californian (Cal), ISSR 4: Papillon (Pap), ISSR 5: Rex (Rex). M =1 Kb marker.

## 3 Results and discussion

In this current investigation, the SCoT primer was utilized for the first time to study the polymorphism and genetic variation within and between several rabbit breeds reared in Egypt. This study aims to estimate genetic diversity among and within rabbit breeds using SCoT and ISSR markers to improve breeding strategies and, moreover, to evaluate the efficiency of markers used in distinguishing genetic diversity among studied breeds. DNA patterns produced by the use of six SCoT primers and seven ISSR primers to analyze the genomic DNA of the five rabbit breeds are shown in Figs. 1 and 2.

### 3.1 SCoT-PCR polymorphism analysis

The six SCoT (Sc 2, Sc 19, Sc 6, Sc 3, Sc 14, and Sc 33) scored 60 bands in total. The number of bands varied from 5 bands with Sc 14 to 13 bands with Sc 33, having an average of 10 bands per primer and various molecular weights ranging from 260.483 to 1610.702 bp.

The bands were found to be dispersed as 47 polymorphic and 13 monomorphic bands, having a high polymorphism average of 78.33 %. Polymorphic bands varied from 13 bands with Sc 33 to 3 bands with Sc 14 in an average of 10 bands per primer. Varying SCoT primers were able to produce sufficient numbers of unique bands (22 positive and 2 negative), differentiating numerous specific markers of the rabbit breeds under investigation. The primers with the highest level of polymorphism were Sc 33 with 92.31 % and Sc 19 with 81.25 % percentage. The primer Sc 14 displayed the lowest degree of polymorphism, with a percentage of 60 % (Table 3).

To the best of our knowledge, this is the first study to investigate the genetic diversity among studied rabbit breeds using SCoT marker. Therefore, there is no previous data on using SCoT markers with rabbits to compare to. It was reported that SCoT analysis was successful in identifying genetic polymorphism in different species. In camels, Al-Soudy et al. (2018) showed that total polymorphism scored by different SCoT primers was (49 %) among four camel breeds, also in Mullet species, total polymorphism was 86.9 %. In chironomids of Manipur, total polymorphism was 91.67 % (Devi et al., 2019), and it was 57 % in gastropod species (Abu Almaaty, 2020).

**Table 4:** Diversity index between the five studied rabbit breeds derived from the SCoT-PCR analysis.

Rabbit breeds	Matrix file input			
	NZW	Cal	Pap	Rex
Gab	4.583	4.000	4.690	5.568
NZW		3.873	4.359	4.472
Cal			4.243	5.196
Pap				4.583

Gab: Gabali breed, NZW: New Zealand white breed, Cal: Californian breed, Pap: Papillon breed, Rex: Rex breed.

**Figure 3:**
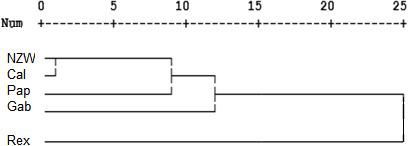
Display of a dendrogram using SCoT-PCR results among the five rabbit breeds.

### 3.2 SCoT similarity matrix and dendrogram

A similarity matrix and dendrogram (Fig. 3 and Table 4) displayed the genetic diversity among the five rabbit breeds that were studied using six SCoT markers. The Rex breed showed the highest diversity when combined with the other four studied breeds at 5.568, with the Gab breed, followed by 5.196 with the Cal breed.

These results suggest that the Rex breed is genetically diverse from the other four breeds. The lowest diversity was recorded between NZW and Cal breeds, 3.873, followed by the diversity between Gab and Cal, 4.000, indicating a shared genetic ancestry and a possible close genetic relationship between those breeds. Based on the previous results from the SCoT-PCR analysis, it is supposed that SCoT-PCR is capable of detecting a high degree of genetic variability and polymorphism.

Dendrogram investigation was conducted based on the presence or absence of patterns using the SCoT primers, and the five rabbit breeds were grouped into three clusters (Fig. 3). Cluster A comprised three breeds (NZW, Cal, and Pap) exhibiting a low level of genetic diversity. In contrast, there is just a single breed (Gab) in cluster B. C is the third cluster, which contains a single breed (Rex). The data suggest that the Rex breed has the highest level of genetic diversity.

These findings were similar to the results of Mohamed and Abdelfattah (2018b), who found that California and New Zealand were more genetically related using different molecular markers (RAPD and SRAP). Also, Galal et al. (2013) concluded that both California and NZW had low genetic variation using ISSR markers.

### 3.3 ISSR-PCR polymorphism analysis

Among the set of 10 ISSR primers, only 7 were able to identify genomic DNA fragments, as illustrated in Fig. 2. In total, 56 bands were acquired using seven ISSR primers to examine diversity within five rabbit breeds. The number of bands per primer ranged from 6 bands in ISSR 2 and ISSR 5 to 10 in ISSR 1; their molecular weights varied from 171.575 to 2418.244 bp, with an average of eight bands per primer.

**Table 5:** Gained bands using seven ISSR primers in five rabbit breeds – total, monomorphic, polymorphic, and unique bands.

Primer	Code	Total no. of bands	Monomorphic bands	Polymorphic bands		Polymorphism (%)
				Without unique	Unique	
ISSR 1	ISSR825	10	4	4	2	60
ISSR 2	UBC811	6	2	4	0	66.667
ISSR 3	UBC808	10	7	3	0	30
ISSR 4	UBC901	8	2	5	1	75
ISSR 5	UBC814	6	3	3	0	50
ISSR 6	UBC826	7	7	0	0	0
ISSR 7	UBC827	9	3	5	1	66.667
Total		56	28	24	4	49.762

**Figure 4:**
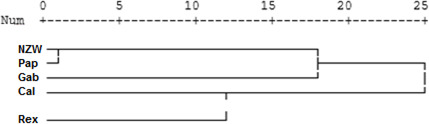
Display of a dendrogram using ISSR-PCR results among the five rabbit breeds.

**Figure 5:**
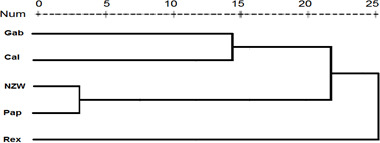
Display of a dendrogram using pooled data from of SCoT–ISSR PCR analysis among the five rabbit breeds.

A total of 28 polymorphic bands were identified, varying from zero bands with the ISSR 6 primer to six bands with primers ISSR 1, ISSR 4, and ISSR 7, with an average of four; the overall average polymorphism rate was 49.762 %. A total of 11 unique bands, consisting of four positive and seven negative bands, were produced by the utilization of seven ISSR primers. With 75 % polymorphism, ISSR 4 was the most polymorphic primer. With the ISSR 6 primer, no polymorphism was scored (Table 5). In a study to evaluate genetic diversity among four rabbit breeds using ISSR primers, DNA fragments were polymorphic in 61.7 % of the cases (El-Sabrout and Aggag, 2015). Molecular markers including ISSR represent reliable tools that may have a great impact on rabbit breeding programs and the genetic improvement of rabbits. Also, ISSR-PCR is a useful method to detect different genetic expressions and understand the variability in some productive traits in rabbits (El-Sabrout and Aggag, 2015).

### 3.4 ISSR–similarity matrix and dendrogram

The similarity index quantifies the diversity and relationship within the five examined rabbit breeds by employing seven ISSR primers as shown in Table 6 and Fig. 4. The highest level of diversity was obtained between Cal and NZW, with a value of 4.243, followed by the diversity between Pap and Rex, with a value of 4.123. The lowest diversity was obtained between NZW and Pap, with a value of 3.000, followed by 3.317 between Gab and NZW.

The results demonstrated that the five examined breeds had comparatively limited genetic variability considering the previous data, which showed an overall polymorphism ratio of 49.762 %, acquired through the ISSR-PCR technique. This could be attributed to their shared genetic ancestry and the potential for high genetic relatedness among these animals.

**Table 6:** Similarity index among the five rabbit breeds based on ISSR-PCR analysis.

Rabbit breeds	Matrix file input			
	NZW	Cal	Pap	Rex
Gab	3.317	3.873	4.000	3.606
NZW		4.243	3.000	3.742
Cal			3.873	3.464
Pap				4.123

Gab: Gabali breed, NZW: New Zealand white breed, Cal: Californian breed, Pap: Papillon breed, Rex: Rex breed.

A dendrogram was assembled by a hierarchical clustering analysis, which depended on the presence and absence of bands. The ISSR marker analysis clustering test grouped the five rabbit breeds into three clusters, as shown in Fig. 4. Two breeds, NZW and Pap, are included in the first cluster (A). On the other hand, cluster B contains one breed, i.e., Gab. The remaining two breeds, Cal and Rex, are part of the third cluster (C). These findings suggested that there may be genetic relatedness and genetic pool sharing between these breeds.

The study found that the ISSR markers demonstrated moderate levels of genetic variability within the examined breeds. These results are in agreement with many studies that stated the usefulness of ISSR markers for detecting genetic diversity among rabbit breeds in Egypt (El-Sabrout and Aggag, 2015) and in Nigeria (Omotoso et al., 2019) and also among other animals, such as snake melons (Ibrahim et al., 2020) and camels (Al-Soudy et al., 2018)

Galal et al. (2013) stated that the genetic variation in domestic breeders allows for the evolution of new features for environmental changes or adaptation to diseases. El-Sabrout and Aggag (2015) used ISSR to genetically determine the genetic similarity and diversity in four rabbit breeds in Egypt. El-Sabrout and Aggag (2015) reported that the local breeds were distinctly different from the Cal and NZW breeds. They expected that because the local and California breeds were genetically far apart, the crossbreeding between them would be suitable for developing new characteristics to improve the productive performance of rabbits.

**Table 7:** Molecular weights of specific unique SCoT – ISSR positive and negative bands that obtained by utilizing six SCoT primers and seven ISSR-primers with five rabbet Breeds.

Gab			NZW			Cal			Pap			Rex		
Primer	MW unique bands		Primer	MW unique bands		Primer	MW unique bands		Primer	MW unique bands		Primer	MW unique bands	
	Positive	Negative		Positive	Negative		Positive	Negative		Positive	Negative		Positive	Negative
ISSR 4	1656.373		SCoT 2	433.697		SCoT 19	1412.423		SCoT 19	541.473		SCoT 2	709.011	496.522
							814.2			448.552			510.139	
													392.736	
ISSR 7		1299.612	SCoT 19	1541.056		SCoT 6		1410.924	SCoT 6	346.642		SCoT 19	494.549	
				69.961										
			SCoT 6	371.354		SCoT 14	711.214		SCoT 3	793.398		SCoT 6	1610.702	
							260.483						377.302	
			SCoT 33	1075.794		ISSR 2		599.553	ISSR 7	717.601	805.965	SCoT 3	779.466	
								504.064						
								348.878						
						ISSR 7		636.808				SCoT 33	922.647	
													308.918	
												ISSR 1	557.412	
													1078.731	
												ISSR 5		683.23

MW: molecular weight, Gab: Gabali breed, NZW: New Zealand white breed, Cal: Californian breed, Pap: Papillon breed, Rex: Rex breed.

**Table 8:** Similarity index among the five rabbit breeds based on pooled data of SCoT–ISSR PCR analysis.

	NZW	Cal	Pap	Rex
Gab	5.74	5.57	6.24	6.71
NZW		5.83	5.29	5.83
Cal			5.83	6.32
Pap				6.16

Gab: Gabali breed, NZW: New Zealand white breed, Cal: Californian breed, Pap: Papillon breed, Rex: Rex breed.

### 3.5 ISSR–SCoT specific marker analysis

Using six SCoT primers, a valuable number of unique bands was obtained (22 positive and 2 negative unique bands), while using seven ISSR primers resulted in four positive and seven negative unique bands, identifying several specific markers shared by the breeds under investigation. The Rex breed was the most characteristic breed that was distinguished by the existence of 13 unique bands (11 positive and 2 negative), 10 unique bands with different SCoT markers, and 3 with ISSR markers. The Cal breed was distinguished by the existence of nine unique bands (four positive and five negative), five unique bands with different SCoT markers, and four with ISSR markers. The Pap breed was identified by the existence of six unique bands (five positive and one negative), four unique bands with different SCoT markers, and two with ISSR markers. The NZW breed was recognized by the presence of five unique bands (four positive) and five unique bands with different SCoT markers. The Gab breed was distinguished by the existence of two unique bands (one positive and one negative).

The molecular weights of specific markers obtained from ISSR and SCoT markers for all five studied breeds are shown in Table 7. Specific molecular genetic markers are helpful fingerprints that describe each breed of rabbit and may be used to distinguish across breeds at the molecular level (M. A. Badr et al., 2016, b; Al-Soudy et al., 2018). Consequently, molecular markers may be suitable for use in genetic polymorphism studies and linkage mapping investigations involving rabbits (Schwartz et al., 2007; Hongmei et al., 2008).

### 3.6 Pooled ISSR–SCoT similarity index and dendrogram

From the grouped ISSR–SCoT examination, the highest diversity, 6.71, was scored between Gab and Rex breeds followed by 6.32 between Cal and Rex, reflecting a low genetic relationship and indicating different genetic resources.

The lowest diversity, 5.29, was observed between Pap and NZW followed by 5.57 between Gab and Cal breeds, showing a high genetic relationship.

Dendrogram analysis revealed that the five studied rabbit breeds were divided into three major groups; the first one is A, which contains Gab and Cal breeds, suggesting that these breeds could have sprung from the same ancestors. Cluster B contains the NZW and Pap breeds. The breed Rex is the only one represented in a cluster (C), Exhibiting a high level of genetic variation compared to other breeds (Fig. 5).

Dendrograms produced from the combined data of multiple molecular markers appear to be more effective than those produced from the data of a single marker alone, and the high boot-strapping values, which indicate how robust the clustering is in the dendrograms, supported this (Lai et al., 2012; Abouzaid et al., 2016).

SCoT markers are shown to be better than ISSR markers for evaluating genetic diversity in multiple studies. Moghaieb et al. (2022) found that SCoT markers generated a higher number of species-specific markers compared to ISSR markers in basil species. Also, Le-Ngoc et al. (2022) reported that SCoT markers distinguished among tested animals more properly and reproduced a higher level of genetic diversity compared to ISSR markers in *Camellia dilinhensis* populations.

SCoT markers had higher polymorphism percentage, polymorphic information content, and marker index compared to ISSR markers in flax genotypes (Osman et al., 2021). Additionally, Nosair (2021) found that SCoT markers are more informative in studying genetic diversity within wheat cultivars compared to ISSR markers. Therefore, based on these studies, it can be concluded that SCoT markers were indeed better than ISSR markers in evaluating genetic diversity.

From previous results, we can expect that the crossbreeding between Gabali and other foreign breeds will be more successful in obtaining many benefits from the two genotypes to improve the productive performance of rabbit genotypes and developing new characteristics to become more adapted to environmental changes because of the high adaptation of the native rabbits to the Egyptian conditions, as observed by Meshreky et al. (2005). This is because they are genetically far apart and other four breeds are selected for daily weight gain and more adapted to environmental changes. Knowledge of genetic diversity among rabbit breeds and the relationship between their genetic markers and performance play an important role in the conservation of the genetic resources of rabbits (Wilkinson et al., 2012).

## 4 Conclusions

The results of this investigation may aid in managing the germplasm, improving present breeding methods, and introducing new crossbreeds. Overall, the features of polymorphism derived from SCoT-PCR between genomic DNA of various breeds of rabbits show highly informative SCoT markers, suggesting the ability to identify the genotype of each animal and determine the phylogenetic relationship. Therefore, DNA polymorphism of different breeds of rabbits detected using SCoT analysis characterized genetic diversity and phylogenetic relationships of these animals. It was established that the SCoT markers may be more effective than ISSR for differentiating and identifying the breeds of rabbits under study.

## Data Availability

The data presented in this study are available free of charge for any user upon reasonable request from the corresponding author.
